# State of pneumococcal vaccine immunity

**DOI:** 10.1080/21645515.2024.2336358

**Published:** 2024-04-03

**Authors:** Mustafa Akkoyunlu

**Affiliations:** Division of Bacterial Allergenic and Parasitic Diseases, Center for Biologics Evaluation and Research, US Food and Drug Administration, Silver Spring, MD, USA

**Keywords:** Pneumococcal vaccines, neonates, TACI, Tfh cells

## Abstract

Like the other invasive encapsulated bacteria, *Streptococcus pneumoniae* is also covered with a polysaccharide structure. Infants and elderly are most vulnerable to the invasive and noninvasive diseases caused by *S. pneumoniae*. Although antibodies against polysaccharide capsule are efficient in eliminating *S. pneumoniae*, the T cell independent nature of the immune response against polysaccharide vaccines renders them weakly antigenic. The introduction of protein conjugated capsular polysaccharide vaccines helped overcome the weak immunogenicity of pneumococcal polysaccharides and decreased the incidence of pneumococcal diseases, especially in pediatric population. Conjugate vaccines elicit T cell dependent response which involve the interaction of specialized CD4+ T cells, called follicular helper T cells (Tfh) with germinal center B cells in secondary lymphoid organs. Despite their improved immunogenicity, conjugate vaccines still need to be administered three to four times in infants during the first 15 month of their life because they mount poor Tfh response. Recent studies revealed fundamental differences in the generation of Tfh cells between neonates and adults. As the portfolio of pneumococcal conjugate vaccines continues to increase, better understanding of the mechanisms of antibody development in different age groups will help in the development of pneumococcal vaccines tailored for different ages.

## Introduction

*Streptococcus pneumoniae* is a gram-positive encapsulated bacteria that causes invasive diseases such as bacteriemia and meningitis as well as noninvasive diseases such as pneumonia and otitis media.^1^ Like the other encapsulated strains, the capsule is composed of a variety of polysaccharide structures that also help the bacteria evade host immune system.^1–3^ There are at least 100 serotypes identified based on the capsular polysaccharide (CPS) structures.^4^ Although the capsule manifests virulence properties, once developed, antibodies against the CPS are efficient in mediating killing *S. pneumoniae* through primarily opsonophagocytic activity.^5^ The recognition of bacterial susceptibility to antibody mediated killing led to the development of vaccines composed of CPS.^6^ These early versions of the pneumococcal vaccines were prepared by purifying CPSs from culture media. The CPS vaccines were able to elicit IgG antibodies against the CPS beyond two years of age and were documented to prevent invasive pneumococcal disease, especially in aged population (IPD).^7^ Although the CPS vaccines are able to induce IgG antibodies, the durability of B cell memory against the CPS is questionable.^8^ Moreover, there are indications for further blunting of vaccine specific B cell memory response upon reimmunization,^9^ although this phenomenon was not observed in every study.^10^

### T cell independent immune response to pneumococcal CPS vaccines

**Figure 1.**
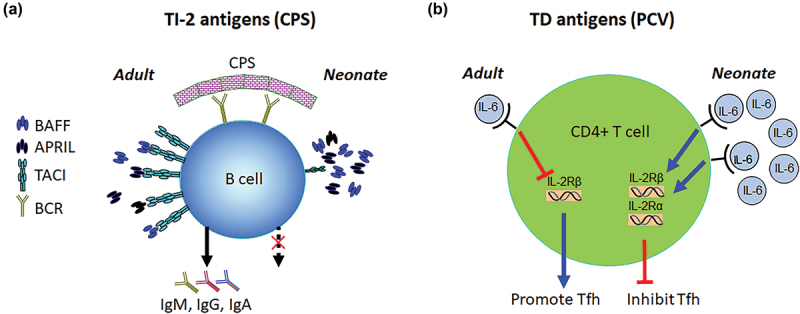
Mechanisms of antibody development against unconjugated (a) and conjugated (b) polysaccharide vaccines. (a) in adults, the recognition of multivalent CPSs by BCR on B cells together with BAFF and APRIL mediated signaling through TACI leads to immunoglobulin secretion. In neonates, CPS recognition by BCR is not sufficient to activate B cells because severely reduced expression of TACI on B cells deprives them from the second signal. (b) in adults, IL-6 promotes tfh generation by decreasing the expression of IL-2 Rβ, which spares tfh cells from IL-2 mediated suppression. In sharp contrast to adults, not only neonates harbor more IL-6 after vaccination, but also IL-6 stimulates the expression of IL-2 Rα and IL-2 Rβ on tfh cells, thereby rendering them susceptible to IL-2 mediated suppression.

The underlying reasons for the shortcomings of CPS vaccines primarily lie in the mechanism of immune response development against the sugar moieties. Unlike the response against protein antigens, response to carbohydrates does not involve the recognition of carbohydrates by T cells with the exception of zwitterionic polysaccharides.^11,12^ As such, immune response to carbohydrates is characterized as T cell independent (TI). Multivalent repeating units of carbohydrates allow the engagement of multiple antigen specific B cell receptors (BCR) on carbohydrate specific B cells. This binding of multiple antigenic epitopes leads to the crosslinking of BCRs, which in turn results in the activation of carbohydrate specific B cells.^12^ There are two types of TI antigens: TI type 1 (TI-1) and TI type 2 (TI-2). Lipopolysaccharides are an example of TI-1 antigens. Here, in addition to the recognition of O-antigen (the repeating oligosaccharide moiety) of LPS molecule by BCR, the same B cells receive a second signal by binding of lipid A molecule to toll-like receptor (TLR) 4. In contrast to TI-1 antigens, the identity of a molecule providing a second signal to BCR engagement by TI-2 antigens (CPS) was thought to be the complement receptor.^12^ However, following the discovery of “the B cell activating factor from the tumor necrosis factor family” (BAFF) system of molecules,^13,14^ the “transmembrane activator and CAML interactor” (TACI) emerged as the second signal to BCR (Figure 1(a)). Together with BAFF receptor (BAFF-R) and B cell maturation antigen (BCMA), TACI is a receptor for BAFF and A proliferation inducing ligand (APRIL), the second ligand of the BAFF system.^15,16^ Studies have revealed that TACI deficient mice^15,16^ or individuals with mutations in *TNFRSF13B*, the gene encoding TACI, cannot mount antibody responses against CPS vaccines.^17^ Supporting the role TACI plays in response to CPS vaccines, we and others have reported that TACI expression is severely reduced in both human and mice newborns and infants^2,18,19^ (Figure 1(a)). We went on to show that neonatal mice that are notoriously unresponsive to the prototype TI-2 antigen, (4-hydroxy-3-nitrophenyl)acetyl-Ficoll (NP-Ficoll) were able to mount robust antibody responses when co-injected with CpG (the TLR9 agonist) and this restoration of immune response was accompanied by CpG mediated upregulation of TACI expression on B cells.^2^ The requirement for a second signal in the induction of antibodies against CPS antigens was further documented by Sen and colleagues who reported that the 23-valent CPS vaccine, Pneumovax23 was able to induce antibody responses because this vaccine was contaminated with TLR agonists. These authors showed that immunization of TLR2 or MyD88 deficient mice failed to mount antibody responses to Pneumovax23 and the removal of the TLR agonists from the vaccine abolished antibody responses against the vaccine serotypes in immunized mice.^20^ Supporting these mouse experiments, transcriptional analysis of whole blood from Pneumovax23 immunized subjects indicated that the signature of genes increased during the first 24 hours of immunization overlapped with the signature of genes induced in vitro by TLR agonists from whole blood.^6^

### T cell dependent immune response to conjugate pneumococcal vaccines

The weak immunogenicity of the CPS vaccines was overcome with the discovery of conjugate CPS vaccines. Following the initial observation that conjugation of carbohydrate molecules with proteins alters the immunogenicity of the carbohydrate moieties by Avery and Goebel in 1929,^21^ efforts to apply this technique to modern vaccines^6^ led to the development of first conjugate vaccines composed of *Haemophilus influenzae* CPS, polyribosyl ribitol phosphate (PRP), and diphtheria toxoid (D).^22^ The conjugate vaccines induced improved immune response against PRP compared to PRP alone and this improved immune response was dependent on the participation of CD4+ T cells.^23^ The conversion of PRP to a T cell dependent (TD) antigen by conjugating to a protein carrier led to the development of other CPS conjugate vaccines against a variety of encapsulated bacteria, including *S. pneumoniae*.^7^ A major advantage of CPS conjugate vaccines has been the ability of these vaccines to elicit protective immune responses in infants.^6,24^ The introduction of both the *H. influenzae* and pneumococcal vaccines in the infant immunization schedule has led to a sharp decrease of morbidity and mortality due to these pathogens.^25,26^ In addition, the establishment of correlates of protection based on serum IgG antibody levels measured in ELISA facilitated the development of additional *H. influenzae*^27^ and pneumococcal vaccines,^28^ which have been especially important for vaccines against *S. pneumoniae* infections because of the need to expand the valency of these vaccines.

Although not experimentally documented, the participation of CD4+ T cells in the response to CPS were initially thought to be a bystander effect of the carrier specific CD4+ cells.^29^ Challenging this hypothesis, Avci and colleagues reported group B streptococcus type III (GBS III) CPS specific CD4+ cells in ovalbumin conjugated GBS III CPS vaccinated mice.^30^ They showed that antigen presenting cells process the protein carriers and present oligosaccharide bound peptides in MHCII. The recognition of peptide bound oligosaccharides by T cell receptor (TCR) leads to the activation of oligosaccharide specific CD4+ T cells.^6^

How different carrier proteins in conjugate CPS vaccines impact the activation of CD4+ T cells and the antibody responses to CPS are not well investigated. Typically, proteins that have been previously used as vaccine components in humans are selected as carrier proteins, mostly because of their favorable safety profiles. For example, diphtheria toxoid is the carrier protein for the first conjugate vaccine, PRP-D.^31^ Similarly, tetanus toxoid (TT) has also been used as a carrier for *H. influenzae* and *S. pneumoniae* conjugate vaccines.^31,32^ The nontoxic variant of diphtheria toxin isolated from cultures of *Corynebacterium diphtheriae* strain C7 (CRM_197_) is used in the first pneumococcal conjugate vaccine (PCV), Prevnar.^32^ Subsequent versions of Prevnar, Prevnar13 and Prevnar20 are also conjugated to CRM_197_.^32^ Other PCV where CRM_197_ is used as a carrier are Serum Institute of India’s 10-valent PCV and Merck’s 15-valent PCV, Vaxneuvance.^32^ There are exceptions to the commonly used carriers that have been used in other vaccines. For example, meningococcal outer membrane proteins were used as a carrier for the *H. influenzae* PRP conjugate vaccine, PedvaxHIB.^31^ Similarly, the 10-valent PCV, Synflorix contains the nontypeable *H. influenzae* (NTHi) outer membrane protein, protein D as a carrier for eight of the ten serotypes in the vaccine.^33^ The two remaining serotypes in 18C and 19F are conjugated to TT and diphtheria toxoid, respectively. Protein D was likely selected as a carrier protein in Synflorix with an expectation to elicit protection against otitis media caused by NTHi in addition to the diseases caused by *S. pneumoniae* primarily because it is highly conserved among NTHi strains and antibodies against NTHi are bactericidal.^34,35^ Although the protective potential of Synflorix against otitis media caused by NTHi was observed in one study with an 11-valent version of Synflorix, the vaccine is not indicated against NTHi mediated otitis media because these favorable results were not replicated in other studies.^33^ The likely reason for Synflorix to not afford protection against otitis media cause by NTHi is because the protein D used in Synflorix is the delipidated version of the native lipoprotein D.^33,34^ Preclinical studies in a rat otitis media demonstrated that the immunogenicity and protective potential of lipoprotein D diminished significantly when lipid moiety is removed.^34^ Similarly, active immunization with lipoprotein D or passive transfer of anti-lipoprotein D antibodies was protective against NTHi in a chinchilla otitis media model.^33^ A more recent example of carrier protein intended to provide benefit beyond engaging CPS specific CD4+ T cells is the investigational 24-valent pneumococcal conjugate vaccine based on multiple antigen presenting system (MAPS) platform.^36^ This investigational vaccine contains a pneumococcal fusion protein composed of two conserved membrane proteins sp1500 and sp0785. The vaccine is intended to extend the vaccine mediated efficacy beyond the 24 serotypes by eliciting opsonophagocytic antibodies as well as T helper 17 response. Pneumococcal membrane proteins also attracted attention as an alternative to conjugate CPS vaccines due to their promise to afford serotype independent protection. The main pneumococcal proteins that have been explored as vaccine against *S. pneumoniae* are pneumolysin, choline-binding protein A, pneumococcal surface protein A, *N*-acetylmuramoyl-L-alanine amidase (LytA), *N*-acetylglucosamindase (LytB), LytC, CbpD, CbpE, CbpF, CbpG, CpbM, pneumococcal histidine triad protein A (PhtA), PhtB, PhtD, PhtE, and pneumococcal serine-rich repeat protein (PsrP).^6^ So far, these vaccines have not advanced beyond phase II trials.

### Germinal center response to conjugate pneumococcal vaccines

As in response to other TD antigens, activated CD4+ T cells upregulate CXCR5 and relocate to germinal centers in secondary lymphoid organs where CXCL13 is expressed following immunization with PCV.^37–39^ These CD4+ cells express the transcription factor Bcl6 and differentiate into follicular T helper cells (Tfh) and interact with germinal center B cells. This interaction leads to somatic hypermutation and affinity maturation of BCR that either become antibody secreting plasma cells or memory B cells. Mouse studies have shown that the generation of Tfh cells during response to TD vaccines or infection is regulated by cytokines that promote (IL-6 and IL-21) and limit (IL-2) Tfh expansion.^37^ The augmentation of Tfh response by IL-6 is mediated by the suppression of IL-2 Rβ on Tfh cells which in turn protects Tfh cells from the detrimental signals elicited by IL-2^40^ (Figure 1(b)). These germinal center reaction details have emerged from adult mouse experiments. Unlike adult vaccines, most pediatric vaccines, including the PCVs, have to be administered 3 to 4 times during the first 15 months of life.^41^ The precise underlying reasons for the need to administer multiple doses of vaccines in order to elicit adult-like protective immune response in early age is poorly understood. The generation of Tfh cells and germinal center B cells is weaker in early age^39,42^ and the suboptimal response to TD vaccines in infants is attributed to limited Tfh generation and germinal center B cell development.^43^ Importantly, the details of TD antigen induced germinal center reaction in infants are not known. By using a neonatal mouse model, we studied the role of IL-6 in the generation of Tfh cells and germinal center B cells in response to TT conjugated pneumococcal serotype 14 CPS (PPS14-TT) vaccine. We first observed that neonatal mouse splenic antigen presenting cells increase IL-6 production significantly more than the adult mice do^44^ (Figure 1(b)). Also, immunized neonatal Tfh cells produced more IL-2 and expressed higher levels of its receptors, IL-2 Rα and IL-2 Rβ compared to immunized adult mice Tfh cells (Figure 1(b)). Interestingly, co-injection of PPS14-TT with recombinant IL-6 (rIL-6) further decreased anti-PPS14 antibody responses compared to neonates immunized with PPS14-TT only.^44^ Conversely, and as reported previously for an inactivated influenzae vaccine,^45^ co-injection of rIL-6 with PPS14-TT in adult mice led to higher anti-PPS14 antibodies than those immunized with PPS14-TT only. Accompanying the decrease in antibody responses, there was a decrease in Tfh and germinal center B cell development in rIL-6 co-injected neonatal mice.^44^ Underscoring the suppressive properties of IL-6 in neonatal Tfh generation, immunization of IL-6 knock-out neonatal mice led to a significant increase in Tfh generation and anti-PPS14-TT antibody production compared to immunized wild-type neonatal mice. The fact that IL-6 has totally opposite effect in neonatal and adult germinal center responses (Figure 1(b)) suggests that age should be factored in when selecting adjuvants for TD vaccines, including the pneumococcal PCVs.

PCV immune responses can also be different in age groups beyond infants. For example, the development of Tfh cells were severely reduced in Prevnar13 immunized aged participants compared to young adults.^38^ Interestingly, despite impaired Tfh generation, the aged population mounted higher anti-polysaccharide antibodies than the young adults. However, the young adults mounted significantly higher OPA titers than the aged population, indicating that Tfh response is more relevant for the development of functional antibodies after PCV immunization. There is also a difference in PCV responses between males and females where females mount stronger response.^38^ Another factor that is suggested to influence PCV response in aged population is the abundance of Th1 cells in pre-immune serum. Conversely, higher Th17 and CD61+ Natural Killer cells negatively correlate with stronger response to Prevnar13.

## Conclusions

Despite the success of PCVs in controlling pneumococcal diseases caused by vaccine serotypes, the development of new PCVs with extended serotype-coverage continues, mostly because of the emergence of disease-causing non-vaccine serotypes. Vaccines currently being evaluated in Phase 3 clinical trials include 21- and 24-valent PCVs.^32^ As the vaccine valency increases, strategies to improve the immunogenicity of higher valency PCVs are needed, especially because the increase in vaccine valency results in a decrease in antibody responses against vaccine serotypes.^46–48^ Suppression due to excessive exposure to the same carrier is proposed as a mechanism for diminished response to serotypes when the serotype coverage expands.^49^ Carrier suppression was initially observed with polysaccharide vaccines against different bacteria conjugated to the same carrier. For example, coadministration of PRP and pneumococcal vaccines with TT as carriers elicited reduced antibody responses against PRP compared to diphtheria conjugated PRP vaccine coadministered with the TT conjugated pneumococcal vaccines.^50^ Similarly, response against PRP after the coadministration of *Neisseria meningitidis* group B outer membrane protein complex (OMPC) conjugated PRP vaccine and OMPC conjugated pneumococcal vaccine was lower when compared to the response after PRP-Cross reactive material 197 (CRM_197_) and OMPC conjugated pneumococcal vaccines were coadministered.^51^ According to the carrier suppression hypothesis, higher frequency of carrier specific B cells likely compete out the lower frequency of polysaccharide specific B cells and impair the activation of polysaccharide specific humoral response.^49^ Despite the global use of PCVs as part of routine vaccination schedules and the expansion of serotype coverage in new vaccines, the exact mechanism(s) responsible for the decrease in antibody responses remains unknown. The need to delineate the reasons for the decrease in antibody responses as the valency of PCVs increase is most relevant for infants and aged population whose responses to current PCVs are markedly lower than the responses of adult vaccinees. Further research in better understanding the molecular and cellular pathways involved in the development protective antibody responses against *S. pneumoniae* will help identify adjuvants that can target these pathways.
